# *Mycoplasma genitalium* Antimicrobial Resistance in Community and Sexual Health Clinic Patients, Auckland, New Zealand

**DOI:** 10.3201/eid2602.190533

**Published:** 2020-02

**Authors:** Anna Vesty, Gary McAuliffe, Sally Roberts, Gillian Henderson, Indira Basu

**Affiliations:** Auckland City Hospital, Auckland, New Zealand (A. Vesty, G. McAuliffe, S. Roberts, G. Henderson, I. Basu);; Labtests, Auckland (G. McAuliffe)

**Keywords:** Mycoplasma genitalium, genotypic antimicrobial resistance, quinolone resistance–determining region, sexually transmitted infections, bacteria, bacterial infections, New Zealand, antimicrobial resistance

## Abstract

Our retrospective study compared genotypic antimicrobial resistance in *Mycoplasma genitalium*–positive specimens collected from 48 community and 33 sexual health clinic (SHC) patients. Macrolide resistance was similar in community (75%) and SHC (76%) patients. We observed no significant difference in fluoroquinolone resistance between community (19%) and SHC (27%) patients (p = 0.66).

Management of *Mycoplasma genitalium* infections is challenging because the limited treatment options have been affected by rapidly evolving resistance to antimicrobial drugs. Molecular approaches are the preferred method of *M. genitalium* detection, and resistance is determined genotypically. 23S rRNA mutations are associated with macrolide resistance and azithromycin treatment failures (*1*–*3*), whereas fluoroquinolone resistance is associated with mutations in the quinolone resistance–determining region, specifically in the *gyrA* and *parC* genes (*4*).

Azithromycin is the first-line treatment for *M. genitalium* infections in New Zealand; second-line treatment relies on moxifloxacin, a fluoroquinolone. A high proportion (72%) of macrolide resistance has been reported in sexual health clinic (SHC) patients in our region (*5*), and elsewhere in New Zealand fluoroquinolone resistance is reported in 23.3% of *M. genitalium*–positive specimens from SHC attendees (*3*), consistent with the high prevalence of macrolide and fluoroquinolone resistance in the Asia–Pacific region (*1*,*3*,*6*). 

## The Study

We performed a retrospective study of all specimens referred to the Microbiology Department at Auckland City Hospital (Auckland, New Zealand) for *M. genitalium* testing in 2017. Referral sites were predominantly general practices in Auckland and SHCs in the Auckland, Northland, and Waikato regions. Ethics approval was granted by the Health and Disability Ethics Committee (approval no. 16/CEN/188). 

DNA had been extracted from specimens following a diagnostic workflow and stored at −80°C. We retrieved DNA samples that tested positive for *M. genitalium* using real-time PCR (*5*,*7*) for this study. We detected 23S rRNA mutations at nucleotide positions 2058 and 2059 (*Escherichia coli* numbering) by using the commercially available PlexPCR kit ResistancePlus MG (SpeeDx, https://plexpcr.com) (*5*) and *gyrA* and *parC* mutations by using PCR amplification of *M. genitalium* nucleotides 172–402 of *gyrA* and 164–483 of *parC* (*8*), followed by sequencing on the Applied Biosystems 3130xl sequencer (Life Technologies, https://www.thermofisher.com). Sequences were aligned against *M. genitalium* reference genes (GenBank accession nos. CP003773 for *gyrA* and *parC* for U25549) by using SeqMan II (DNASTAR, https://www.dnastar.com) and the mutations reported by using *M. genitalium* numbering. We compared prevalence of resistance-associated mutations in community and SHC cohorts by using a χ^2^ test (α = 5%).

We tested 302 clinical specimens from 247 patients; 33% (101/302) of samples from 34% (84/247) of patients were *M. genitalium *DNA–positive. Four samples from 3 patients were excluded from subsequent analyses because insufficient PCR products were obtained for sequencing. We used the remaining 97 *M. genitalium *DNA–positive samples obtained from 81 patients to determine macrolide and fluoroquinolone resistance. Specimens were urine samples (92%) or urogenital swabs. The mean (+ SD) age of patients was 29 (+ 7.5) years, and 80% (65/81) of patients were men; 59% (48/81) were community patients, and 41% (33/81) were SHC patients.

We detected macrolide mutations in 75% (61/81) of patients (Table 1). We observed no significant difference in the prevalence of macrolide resistance between community (75% [36/48]) and SHC (76% [25/33]) patients (p = 1.00). Fluoroquinolone-resistant strains of *M. genitalium* were identified in 22% (18/81) of patients on the basis of the presence of mutations previously associated with phenotypic antimicrobial resistance or treatment failure (Table 2). We observed no significant difference in proportions of fluoroquinolone resistance between community (19% [9/48]) and SHC (27% [9/33]) patients (p = 0.66).

**Table 1:** Prevalence of antimicrobial resistance in *Mycoplasma genitalium* strains from community and SHC patients, Auckland, New Zealand, 2017*

Genotypic-resistance profile					Frequency, %		
	Mutation				Community patients	SHC patients	All patients
	23S rRNA	*parC*	*gyrA*				
Wild type	–	–	–		20.8	24.2	22.2
Macrolide	+	–	–		60.4	48.5	55.6
Macrolide + fluoroquinolone	+	+	–		12.5	12.1	12.3
Macrolide + fluoroquinolone	+	+	+		2.1	15.1	7.4
Fluoroquinolone	–	+	–		4.2	0	2.5

*SHC, sexual health clinic; +, positive; –, negative.

**Table 2:** Position and number of patients with mutations detected in the quinolone resistance–determining regions of the *gyrA* and *parC* genes in *Mycoplasma genitalium* strains from community and SHC patients, Auckland, New Zealand, 2017*

Gene and mutation†	Amino acid change	No. patients with mutation			
		Community patients	SHC patients	All patients	References
*gyrA*					
G285A	Met → Ile (95)	1	4	5	(*1**,**9*)
G285T	Met → Ile (95)	0	1	1	(*11*)
*parC*					
C184T‡	Pro → Ser (61)	3	2	5	(*1**,**9**,**11*)
G241T	Gly → Cys (81)	1	0	1	(*1**,**12*)
A247C	Ser → Arg (83)	1	0	1	(*1**,**3**,**9**,**12*)
G248T	Ser → Ile (83)	2	6	8	(*1**–**3**,**9*)
T249A	Ser → Arg (83)	1	0	1	
G259A	Asp → Asn (87)	2	0	2	(*1**–**3**,**8**,**9**,**11*)
G259T	Asp → Tyr (87)	2	3	5	(*1**,**2**,**8**,**10*)
C356T‡	Ala →Val (119)	1	0	1	(*11*)

*Nucleotide position and amino acid changes shown are based on *M. genitalium* numbering. SHC, sexual health clinic. †Silent mutations not reported. ‡Fluoroquinolone resistance not determined.

Missense mutations in *parC* at amino acid positions 81, 83, or 87 conferred fluoroquinolone resistance (Table 2). Mutations in codon 83 of *parC* are associated with resistance; therefore, the mutation at T249A was presumed to confer fluoroquinolone resistance. The importance of polymorphisms in *parC* at C184T and C356T is uncertain. Mutations in *gyrA* at codon 95 were detected in 6 patients, 5 of whom were SHC attendees. All 6 patients harbored concomitant *parC* mutations that are associated with fluoroquinolone resistance, meaning that mutations in *gyrA* were not detected in the absence of *parC* mutations. The 5 patients with mutations in *gyrA* at G285A harbored a concurrent *parC* mutation at G248T, suggesting the strains were similar.

We detected dual macrolide and fluoroquinolone resistance in 20% (16/81) of patients (Table 1). Fluoroquinolone-resistant strains were likely to show concurrent macrolide resistance (89%), with the exception of strains from 2 community patients, which harbored only a fluoroquinolone resistance–associated mutation at codon 83 in *parC*.

Repeat specimens from 2 community patients were suggestive of macrolide resistance developing during the sampling period. For 1 patient, 2 of 3 urine samples received at 2-month intervals were negative for 23S rRNA mutations, with a mutation only detected in the most recent of the 3 samples. Another community patient was infected with a strain that harbored a *parC* mutation in codon 83 only; however, a 23S rRNA mutation was later detected in 2 subsequent urine samples collected at 1-month intervals, and the *parC* mutation persisted. A SHC attendee harbored both 23S rRNA and *parC* resistance–associated mutations initially, and a subsequent sample collected 5 months later harbored a mutation in *gyrA* at G285T in addition to the 23S rRNA and *parC* mutations.

## Conclusions

Our findings imply that resistance is common in circulating *M. genitalium* stains in the general population and highlight the limited and declining antimicrobial options for treatment. Few studies have delineated antimicrobial resistance by referrer type, and our results imply that macrolide- and fluoroquinolone-resistant strains are endemic in sexual networks in the general population, rather than confined to, or disproportionately affecting, persons attending SHCs. This information might be useful at local and national levels for informing sexual health treatment guidelines, but a need exists for supranational monitoring and reporting of resistance, given that it varies between countries (*13*).

Infections caused by strains resistant to both macrolides and fluoroquinolones occurred in 20% of patients, who would require alternative treatment options to obtain clinical and microbiological cure. Although pristinamycin has been successfully used to treat patients with multidrug-resistant infections (*14*), this treatment is not publicly funded in New Zealand and is only available as an imported medicine by special approval, underscoring the importance of exploring new treatment strategies to manage patients with resistant strains.

The presence of fluoroquinolone resistance in macrolide-sensitive strains in the community is concerning and might signify emergence of circulating fluoroquinolone-monoresistant strains in the region, with consequent implications for future treatment strategies. Although our data do not distinguish between descendants of clonal mutant lineages and de novo variants, we speculate that the introduction of a resistant clone from overseas is likely and that the use of fluoroquinolones in the community contributes to selective pressure for resistant strains.

A limitation of this study was that we did not have patient information regarding treatment of infection or clinical outcomes. This information might help establish whether resistance developed during treatment or occurred through reinfection with a more resistant strain. Another limitation was the inability to establish chain of transmission between patients, which was particularly relevant to the 4 SHC attendees and 1 community patient who harbored similar strains with identical mutations in both the *gyrA* and *parC* genes.

Mutations in the *gyrB* and *parE* genes act synergistically to increase fluoroquinolone resistance when detected with mutations in *gyrA*, *parC*, or both and might also warrant consideration when screening strains for markers of resistance (*15*). We found that in genotypically fluoroquinolone-resistant strains, single-nucleotide polymorphisms in *gyrA* were only detected with concomitant *parC* mutations. This finding might support diagnostic laboratories in efforts to target only the 23S rRNA and *parC* genes during their genotypic-resistance testing. 
